# Two Lamprey Hedgehog Genes Share Non-Coding Regulatory Sequences and Expression Patterns with Gnathostome Hedgehogs

**DOI:** 10.1371/journal.pone.0013332

**Published:** 2010-10-13

**Authors:** Shungo Kano, Jin-Hua Xiao, Joana Osório, Marc Ekker, Yavor Hadzhiev, Ferenc Müller, Didier Casane, Ghislaine Magdelenat, Sylvie Rétaux

**Affiliations:** 1 Laboratoire Neurobiologie et Développement UPR3294 Centre National de la Recherche Scientifique (CNRS), Institut Alfred Fessard, Gif-sur-Yvette, France; 2 Department of Biology, Center for Advanced Research in Environmental Genomics, University of Ottawa, Ottawa, Canada; 3 Department of Medical and Molecular Genetics, School of Clinical and Experimental Medicine, College of Medical and Dental Sciences, University of Birmingham, Birmingham, United Kingdom; 4 Laboratoire Evolution, Génomes et Spéciation UPR9034 Centre National de la Recherche Scientifique (CNRS), Gif-sur-Yvette, and Université Paris 7, Paris, France; 5 Génoscope, Institut de Génomique, Commissariat à l'Energie Atomique (CEA), Evry, France; Institute of Evolutionary Biology (CSIC-UPF), Spain

## Abstract

*Hedgehog* (*Hh*) genes play major roles in animal development and studies of their evolution, expression and function point to major differences among chordates. Here we focused on *Hh* genes in lampreys in order to characterize the evolution of Hh signalling at the emergence of vertebrates. Screening of a cosmid library of the river lamprey *Lampetra fluviatilis* and searching the preliminary genome assembly of the sea lamprey *Petromyzon marinus* indicate that lampreys have two *Hh* genes, named *Hha* and *Hhb*. Phylogenetic analyses suggest that *Hha* and *Hhb* are lamprey-specific paralogs closely related to *Sonic/Indian Hh* genes. Expression analysis indicates that *Hha* and *Hhb* are expressed in a *Sonic Hh*-like pattern. The two transcripts are expressed in largely overlapping but not identical domains in the lamprey embryonic brain, including a newly-described expression domain in the nasohypophyseal placode. Global alignments of genomic sequences and local alignment with known gnathostome regulatory motifs show that lamprey *Hhs* share conserved non-coding elements (CNE) with gnathostome *Hhs* albeit with sequences that have significantly diverged and dispersed. Functional assays using zebrafish embryos demonstrate gnathostome-like midline enhancer activity for CNEs contained in intron2. We conclude that lamprey *Hh* genes are gnathostome Shh-like in terms of expression and regulation. In addition, they show some lamprey-specific features, including duplication and structural (but not functional) changes in the intronic/regulatory sequences.

## Introduction

Lampreys and hagfish are the only two groups of agnathans (meaning jawless vertebrates) that have survived to date. They belong to a monophyletic group, the cyclostomes, considered as the sister group of extant gnathostomes (or jawed vertebrates) [1]–[3], see also [4]. They are promised to be a first-class model for the study of the evolution of vertebrate developmental mechanisms, owing to this key phylogenetic position and to the special anatomical features they present (reviewed in [5]). The rapidly growing number of reports on various particular genes and gene families in several lamprey species together with the ongoing assembly of genome data for the marine lamprey *Petromyzon marinus* further give these animals a very crucial status to investigate the origins of the vertebrate genomes, including the genomic and functional outcomes of whole genome duplication events (WGD) [6]–[8].

Hedgehog (Hh) family of intercellular signalling proteins are one of the key mediators of many fundamental processes in embryonic development, and are particularly essential to the development of nervous system in gnathostomes (reviewed in [9], [10]). *Hh*-related genes have been identified in most of the chordates genomes sequenced so far, such as the cephalochordate *Branchiostoma floridae*, the urochordate *Ciona intestinalis* and many vertebrates, although these genomes differ in the number of *Hh* genes they contain. Amphioxus has only one *Hh* gene (*AmphiHh*; [11]) and *Ciona intestinalis* has two, *CiHh1* and *CiHh2* [12], which are likely to result from a lineage-specific duplication event. Three *Hh* genes were identified in tetrapods such as mouse, chick, and human: *Desert hedgehog* (*Dhh*), *Indian hedgehog* (*Ihh*) and *Sonic hedgehog* (*Shh*), with *Shh* and *Ihh* appearing more related to each other than to *Dhh*. Two WGD are most likely at the origin of these paralogous genes. An ancestral *Hh* gene first duplicated and gave rise to *Shh/Ihh* and *Dhh* ancestor genes. An additional duplication event generated *Shh*, *Ihh*, *Dhh* and a fourth gene quickly lost [13], [14] (summarised on Fig. 2, inset). In zebrafish, the Hedgehog family is further enlarged due to a teleost-specific WGD, and there are two *Shh* (*shha* and *shhb*, the latter previously called *tiggy-winkle hh*), two *Ihh* (*ihha* and *ihhb*), but one *Dhh* member.

Vertebrate embryos express *Shh* in key signalling centres from which it is secreted and exerts its so-called morphogen effect: the notochord and the floor plate of the neural tube –together with the zone of polarizing activity (ZPA) of the limb buds [15]. Further functional studies have demonstrated that *Shh* (and *Ihh* to a lesser extend) play an essential role in the dorso-ventral and antero-posterior patterning of the neural tube [16], as well as in the definition of anterior-posterior polarity of the limbs. *Dhh* on the other hand is involved in the development of peripheral nerves (the myelin-forming cells) and is expressed in adult nerves [17].

**Figure 1:**
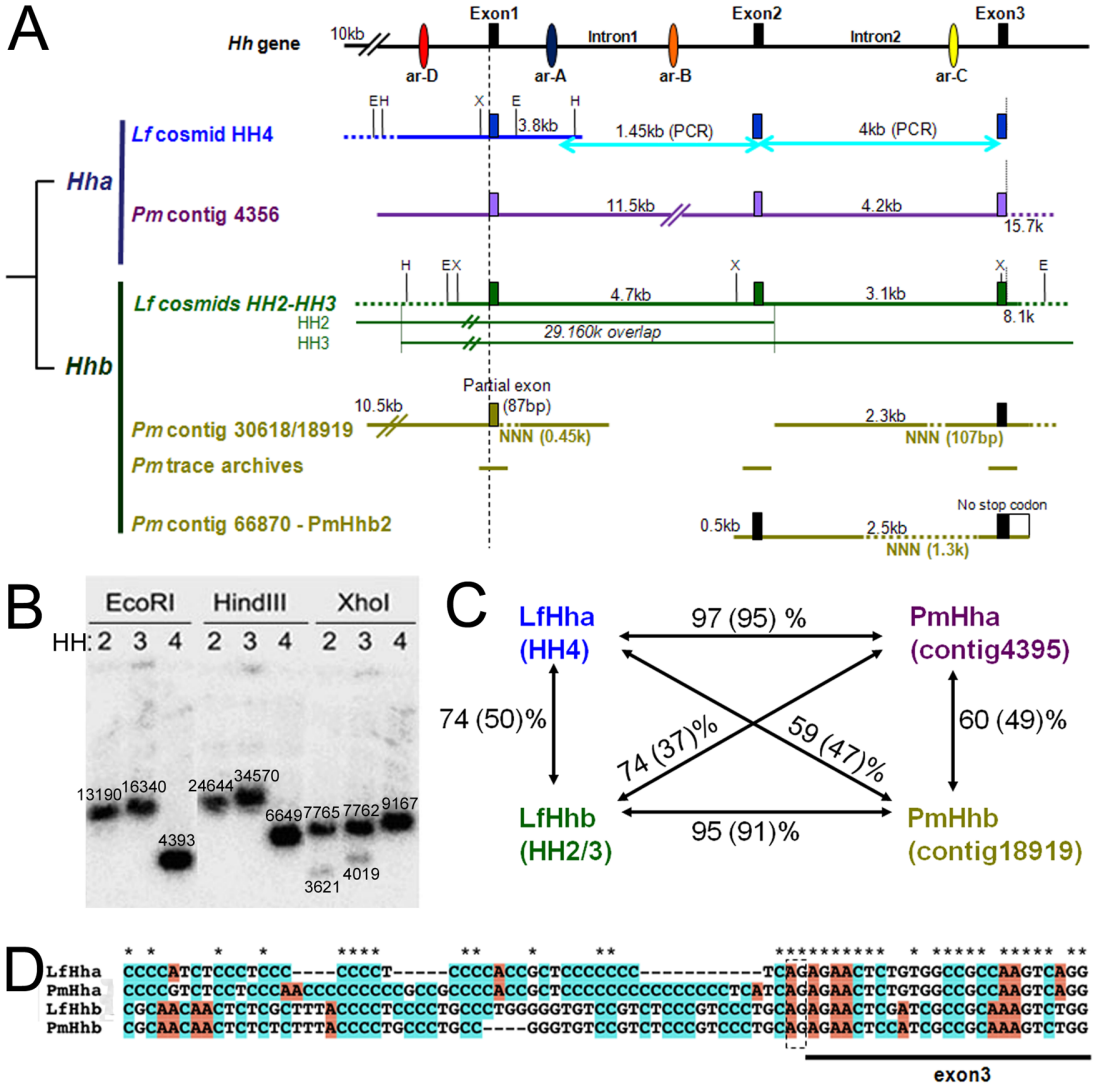
Organisation of *Hha* and *Hhb* loci in *Lampetra (Lf)* and *Petromyzon (Pm)*. **A,** Sequence information available after library screening (*Lf* cosmids), *in silico* searches (*Pm* contigs and trace archives) and PCR amplification is schematically depicted. Drawing is not to scale. The top row shows a “generic” *Hh* gene, with 3 exons (black boxes) and 2 introns containing previously described regulatory elements (coloured ovals, ar-A, B, C, and D; see text). Below, *Hha* genes are drawn in blue/purple, *Hhb* genes are drawn in green/kaki. The sizes of introns and the overall length from ATG to Stop codons are indicated. Restriction sites for enzymes EcoRI (E), HindIII (H) and XhoI (X) used for Southern analysis of the cosmids are indicated (restriction sites located on the Lawrist7 vector are not indicated). The trace archives for putative *PmHhb* exonic sequences have the following IDs (plus and minus are directions for assembly). For exon1: gnl|ti|1427444168+, gnl|ti|1179676842+, gnl|ti|1483498011+, and gnl|ti|1482777522-. For exon2: gnl|ti|1484276315+ and gnl|ti|1482717043+. For exon3: gnl|ti|1470810056+, gnl|ti|1213886743+, gnl|ti|1193744006-, and gnl|ti|1192802884-. **B**, Southern blot of *Lf* cosmids HH2, 3, and 4, using the partial previously isolated *Lf* cDNA encompassing most of exon1 (174 nt) and part of exon2 (100 nt) [23] as a probe (see Figure 2C for probe localisation on cDNA). Restriction enzymes and band sizes (in bp, obtained from *in silico* restriction digest of the HH cosmid sequences) are indicated. **C**, Similarity between each coding region and entire locus (in parenthesis) of the lamprey *Hh* genes. Nucleotide sequences were aligned with the CHAOS/DIALIGN program and the percent identity was calculated using the ClustalX program (ver2.11; [27]). The values show high similarities between the orthologs even when including intronic regions (see the value in the parentheses), while it is much lower between paralogs despite genes of the same species. **D**, An example of alignment showing the high sequence identity between orthologs and the lower sequence identity between paralogs of lamprey *Hh* genes. The sequence shown is at the level of the intron2-exon3 junction. The splice acceptor site (AG) is indicated.

In a one-day-old zebrafish embryo, *Shh* is expressed in the notochord and in nested regions of the central nervous system including the floor plate, the *zona limitans intrathalamica* (zli), the hypothalamus and the retina [18]. Characterisation of regulatory sequences responsible for the spatio-temporal pattern of *Shh* expression identified at least four enhancer regions, ar-A, ar-B, ar-C, and ar-D (where ar- stands for activating region, see Fig. 1 for a general view). Ar-A, B, and C are intronic, whereas ar-D is located in the 5′ untranslated region. In zebrafish, ar-A and ar-C control notochord expression, whereas ar-D and ar-B are responsible for floor plate expression. Ar-A, ar-B and ar-C are together required to drive the expression in the hypothalamus and tegmentum. Ar-C also mediates expression in the zli [19], [20], which is considered as a secondary forebrain organiser. Several of these enhancers are conserved in sequence with other gnathostomes as demonstrated by phylogenetic footprinting, and they are thus called conserved non-coding elements (CNEs). However, functional conservation of these enhancers is not the rule: for example the ar-C element located in intron2 drives notochord and forebrain expression in zebrafish [19], while its mammalian counterpart (called SFPE2 for Sonic Floor Plate Enhancer2) drives floor plate expression in the mouse [21]. Whether lampreys present such sequence and/or functional conservation in *Hh* non-coding regions has not been addressed so far.

Partial Hh cDNA fragments have been isolated for two lamprey species, *Lampetra japonica* [22] and *Lampetra fluviatilis* [23]. *Lampetra fluviatilis Hh* transcripts were detected in the notochord and prechordal plate, the floor plate, the diencephalic zli and a small hypothalamic region, thus strongly resembling the “archetypal” jawed vertebrate *Shh* pattern [23]. Compared to non-vertebrate chordates, lamprey *Hh* expression further extends into anterior parts of the neural tube including the forebrain. Unlike gnathostome *Shh,* however, lamprey *Hh* expression is absent from the preoptic area and from the ventral telencephalon [5], [23], [24]. These differences in expression further suggest a relationship between *Hh* expression domains and the development and evolution of the organisation of the central nervous system [5], [25].

Here, we focus on *Hh* gene(s) in two species, the river lamprey *Lampetra fluviatilis* (*Lf*) and the sea lamprey *Petromyzon marinus* (*Pm*), to better understand the evolution of the Hh signalling system at the emergence of vertebrates. We provide evidence for the existence of two lamprey *Hh* genes, we describe their genomic organisation and compare their expression features, and we characterise a functional midline enhancer shared among vertebrates.

## Materials and Methods

### Ethics statement

Animals were treated according to the French and European regulations for handling of animals in research. SR's authorisation for use of animals in research is number 91–116. This study uses exclusively embryos from aquatic vertebrate (non mammalian) animals and therefore did not require special authorizations.

### Screening and sequence analyses (Fig. 1A)

#### Lampetra library screening

An *Lf* cosmid genomic library (RZPD library 55) was screened using classical molecular techniques, at low stringency, and using the previously isolated *Lf* cDNA fragment [23] as a random-primed DNA radioactively labelled probe. Out of the five positive clones identified, four were confirmed by Southern blot and 5′-end sequencing to be *Hh* positive clones. The full sequence for three of them (HH2, 3, and 4) was obtained after 454 sequencing at the Genoscope Sequencing Centre (Evry, France) and was submitted to GenBank under the accession numbers FP929026 (HH2), FP929027 (HH3) and FP929028 (HH4).

#### Petromyzon in silico searching

*Pm* contigs were retrieved *in silico* after BLAT or BLAST searches on the preliminary assembly of the sea lamprey genome using other vertebrates' *Hedgehog* genes (mouse and zebrafish) and the *Lampetra Hh* loci sequences obtained above as queries on the UCSC genome browser (http://genome.ucsc.edu/cgi-bin/hgGateway?clade=other&org=Lamprey&db=)or the preEnsemble database (http://pre.ensembl.org/Petromyzon_marinus/Info/Index).

Using the *LfHhb* coding sequence as query, best hit sequences were retrieved by blastn searches on the NCBI Trace Archive for *P. marinus* whole genome sequence (http://blast.ncbi.nlm.nih.gov/Blast.cgi). “EST” or “other” did not result in obtaining any significantly similar sequences. The retrieved sequence archives were assembled using the CAP3 program (Huang and Madan, 1999) implemented on the website, Mobyle@Pasteur (http://mobyle.pasteur.fr/cgi-bin/portal.py). Each resulting assembled contig was located on Contig30618, Contig31827, and Contig18919. Coding regions were predicted from the contigs with aids of BLASTX. Every putative exons was distinct from those of *PmHha* located on PmContig4356. Thus, we postulated that these exons belong to a single gene, namely *PmHhb*, although its continuity is not validated.

#### PCR amplifications

Intron2 of *LfHha* was amplified from *Lampetra* genomic DNA with primers Intron2-for (TGGGTCTACTACGAGTCCAAGG) and PmIn2-rev2 (CTTGGCGGCCACAGAGTT), followed by nested PCR using primers LfHhaI2_fwd (GTACGAATACTGGACTGGGATCG) and LfHhaI2_rev (GCAGTGAGCGGACGTTAGAC), and cloned into pGEM®-T Vector (Promega). A positive clone was sequenced with plasmid walking. To ascertain the continuity of the *LfHha* locus, partial exon1-exon2-partial exon3 was amplified from *Lampetra* cDNA obtained previously [23] with primers His_hha_for3 (GTCGCTACGAGGGGAAGAT) and His_hha_rev2 (TTCACGCACGAACACAAAGT), followed by cloning into pGEM®-T Vector and sequencing.

Exons were predicted with BlastX searches and manual editing based on alignment with cDNA sequences and on the canonical splicing rules (see Figure S2B). The nucleotide sequences were aligned with the CHAOS/DIALIGN program and the percent identities between the four identified *Hhs* were calculated using ClustalX (ver2.11) [26], [27].

### Phylogenetic analyses

A multiple sequence alignment was carried out with the ClustalX software [28] and optimized manually using the MUST software [29]. After removing regions of ambiguous homology, an edited alignment of 234 amino acid positions was used in subsequent analyses. We first applied ProtTest [30] to estimate the optimal model of amino acid substitution (LG + Γ + I). Using this model, a maximum likelihood (ML) tree was inferred using PHYML [31]. The robustness of the ML tree was estimated by 100 bootstrap replications. A Bayesian analysis was performed using MrBayes [32] with the optimal closest model implemented in this program (JTT + Γ + I) and the following MCMC parameters: 1,000,000 generations, sampling each 20 generations, 250 “burn-in” trees discarded to reconstruct the consensus tree. Pairwise sequence distances were computed with the JTT + Γ model (alpha parameter, 0.41, estimated with PHYML) and a Neighbor-Joining (NJ) tree was inferred with MEGA software [33]. The robustness of the NJ tree was estimated by 1,000 bootstrap replications.

Accession numbers for Hh proteins included in the phylogenetic tree are as follows: Chicken shh, NP_990152; Human shh, NP_000184;Mouse shh, NP_033196; Xenopus laevis Shh, NP_001081782Leucoraja Shh, ABM66102Zebrafish shhb, NP_571274; Fugu shh, AAT99577; Zebrafish shha, NP_571138; Zebrafish ihhb, NP_571163; Zebrafish ihha, NP_001030165; Fugu Ihh, ENSTRUT00000034629; Fugu Ihhb, ENSTRUT00000031084; Chicken ihh, NP_990288;Xenopus ihh, NP_001079262; Mouse ihh, NP_034674; Human ihh, NP_002172; Fugu Dhh, ENSTRUT00000030985; Xenopus dhh, NP_001079261; Human dhh, NP_066382; Mouse dhh, NP_031883; Zebrafish dhh, NP_001025286; Amphioxus hh, CAA74169; Fruitfly hh, NP_524459.

### Sequences alignments for CNEs searching

The global alignment algorithm LAGAN [34] was used to perform the pair-wise alignments for the whole Hh loci, subsequently visualized with VISTA plot [35], [36].

The multiple local alignments of putative ar-C sequences were obtained using the CHAOS/DIALIGN algorithms [26], and then visualized with BioEdit (written by Tom Hall, Ibis Biosciences, Carlsbad, CA, USA).

### Whole-mount *in situ* hybridization

**Figure 2:**
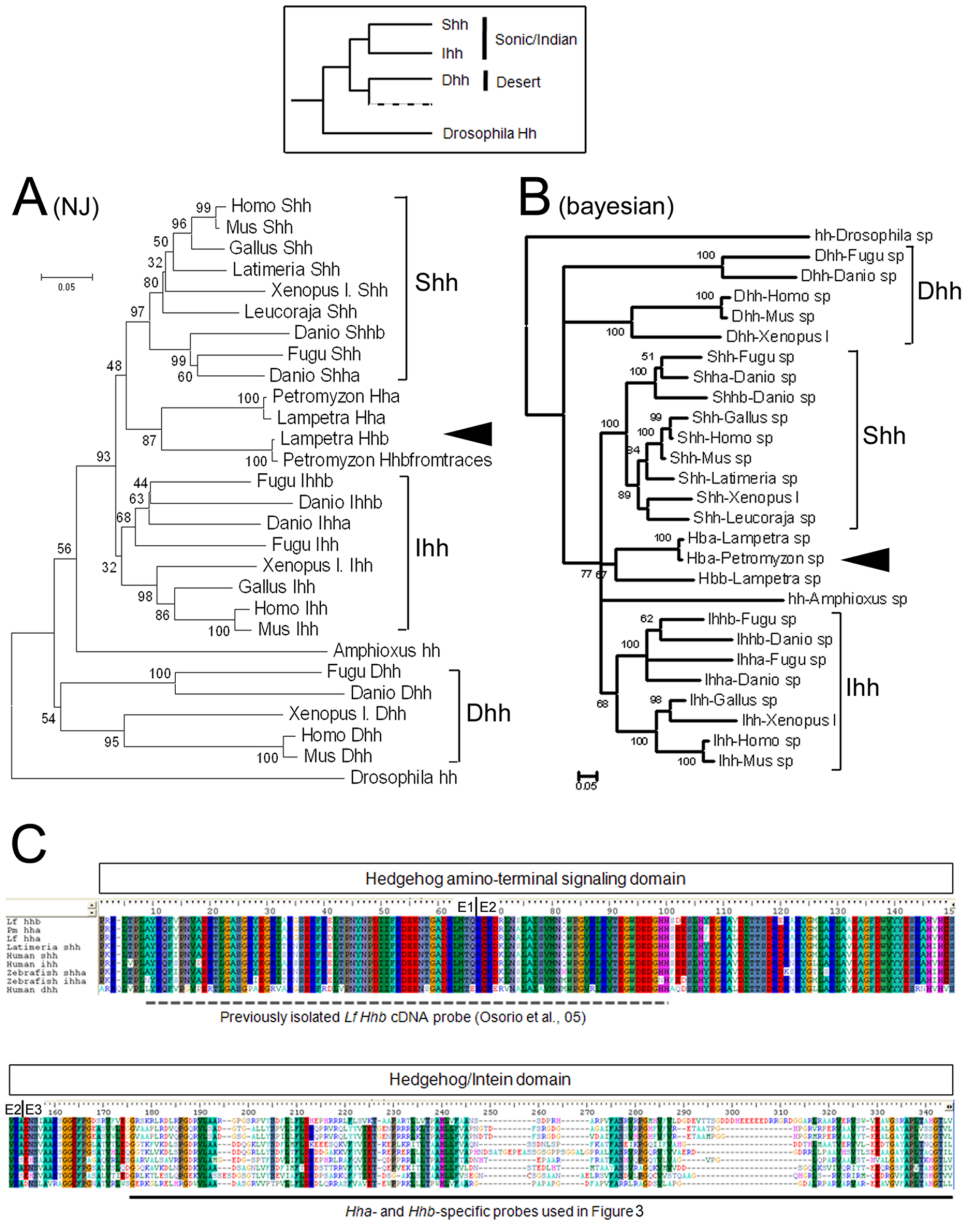
Phylogenetic analysis of lamprey's Hhs. Inset: a minimal representation of the Hedgehog family in vertebrates, to highlight the classically-described relationships between Sonic, Indian, and Desert groups (see text, Introduction). **A** and **B** are Neighbour-Joining (NJ) and Bayesian phylogenetic trees (aligned aminoacids) of 28 (NJ) and 27 (Bayes) Hedgehog family members including the presently found lamprey members (black arrowhead), with the fly and amphioxus Hhs used as out-group. Bootstrap values are given and the 3 orthology groups are indicated on the right (Sonic, Shh; Indian, Ihh; and Desert, Dhh). **C** is an amino-acid alignment of lamprey Hh proteins with gnathostome family members. The functional domains (HH signal and Hint domain) are indicated, as well as the exon (E) junction positions (e., g., E1/E2). The regions corresponding to *Hha*- and *Hhb*-specific *in situ* hybridization probes used in Figure 3 are also indicated, as well as the previously isolated *Lf* probe used in [23].

*LfHha*- and *LfHhb*-specific probes were designed on the third exon of each paralog, amplified from *Lf* cDNA with specific primers, and subcloned into pCR-TOPOII (Invitrogen) (see Fig. 2B for exact location of the specific probes). The following primers were used for amplification: HhaP_m_for (5′-GGC GTC GCC GCT CCG CTG CGA-3′), HhaP_m_rev (5′-CGA GCA CCG TGC CGC TCG ACA C-3′), HhbL_f_for (5′-GGC CGA TCC AAG CGG CTC CG-3′) and HhbL_f_rev (5′-CCA GCG TGC CGT GAG CCG T-3′). Digoxigenin-labeled antisense riboprobes against *Hha* and *Hhb* mRNAs were synthesised and whole-mount *in situ* hybridization was performed on *Lampetra* embryos as previously described [23], [25]. Some embryos were dehydrated with graded ethanol series and cleared with a 1 benzyl alcohol: 2 benzyl benzoate solution for whole-mount observation. Other embryos were dehydrated in ascending ethanol and butanol, embedded in paraffin and sectioned with a Leica microtome at 8 µm. Photographs were taken on a Nikon microscope equipped with a DXM-1200 camera.

### DNA constructs

*LfHhb* intron2 was amplified from the *Lf* cosmid HH3 using a high-fidelity polymerase, AccuPrime™ Taq DNA Polymerase High Fidelity (cat#12346-086, Invitrogen), and ligated into *Kpn*I and *Not*I sites of the backbone vector, −0.8Shh:GFP, which contains the minimal promoter of zebrafish *shha* and a multi-cloning site (MCS) downstream of the GFP reporter [37]. For cloning of a 30 bp fragment of the putative *LfHhb* C1 element, two complementary oligonucleotides were annealed after heat-induced denaturation, and used as an insert fragment for cloning into the same vector. Each primer sequence is designed to introduce the restriction sites as follows, with the annealing sites and additional restriction sites indicated in capital and small letters, respectively: LfHhb-I2_fwd_NotI (aaagcggccgcACTACGAGTCCAAGGCGCAC) and LfHhb-I2_rev_KpnI (cggggtaccCGGCGATCGAGTTCTCTG) for *LfHhb* intron2; LfHhb-C1-Fwd (ggccgcAGGGAATTTCGCACCTGAGCAAACGAGGAGggtac) and LfHhb-C1-Rev (cCTCCTCGTTTGCTCAGGTGCGAAATTCCCTgc) for *LfHhb* putative C1 element.

### Microinjection of zebrafish eggs

Fish care and microinjection were carried out as described in [38]. The concentration of DNA constructs was adjusted to 70 ng/µl, and embryos were raised at 28°C. To precisely score the ratio of positive embryos, immuno-staining against GFP was carried out. Briefly, embryos were fixed in 4% PFA, followed by three washes with PBST. They were incubated with rabbit polyclonal anti-GFP at 1/1000 dilution (Molecular Probes, refA6455) at 4°C overnight, followed by washes and incubation in secondary chicken Alexa488 anti-rabbit antibody (Molecular Probes, refA21441) at room temperature for 2 hours. After PBST washes, they were stored in PBS until observation. The GFP fluorescence was scored and photos were taken using a Macrozoom system (Nikon).

## Results and Discussion

### Two species of lampreys each have two Hedgehog genes

#### Lampetra fluviatilis

Screening of a river lamprey (*Lf)* cosmid genomic library generated several positive clones which were subjected to Southern blot and showed two types of restriction profiles (Fig. 1B), raising the possibility of the existence of two distinct *Hh* genes in *Lf* genome. Three selected cosmids, HH2, HH3 and HH4, were sequenced and analysis revealed two clearly distinct *Lf Hh* loci.

Cosmid HH4 (33.4 kb, including 11.4 kb of upstream sequences containing a putative gene, pol polyprotein-like) did not contain the whole *Hh* locus, spanning the first exon and part of the first intron of an *Lf Hh* gene corresponding to the *Lf* cDNA fragment used as a probe [23]. We named this gene *LfHha* (blue, Fig. 1A). Additional PCR amplifications of the [intron1-exon2] and [exon2-intron2-exon3] regions of *LfHha* using *Lf* genomic DNA as template enabled us to obtain an almost complete sequence for *LfHha* (light blue lines on Fig. 1A). Proof of continuity of the *LfHha* locus and determination of the intron-exon boundaries was further obtained by PCR amplification on *Lf* cDNA template of a partial exon1-exon2-partial exon3 sequence (corresponding to amino-acids 38 (R) to 285 (V) of the LfHha protein sequence shown in Fig. 2C).

The other two cosmids, HH2 and HH3 could be assembled into a 48.3 kb contig, with 29.2 kb of overlapping sequence, including 32.4 kb of upstream sequences, 9.2 kb within the *Hh* locus, and 6.7 kb of downstream sequences. The two cosmids HH2/HH3 encompassed the whole genomic locus of an *LfHh* which was different from *LfHha*, as deduced from both sequence comparisons and Southern analysis (Fig. 1B). It was therefore named *LfHhb* (green, Fig. 1A).

#### Petromyzon marinus

*In silico* searches of the preliminary assembled genome of *Petromyzon marinus* retrieved four hits corresponding to *Pm* contigs 4356, 30618, 18919 and 66870.

Contig 4356 (23.5 kb) contained the almost full sequence of a *Hh* gene that was the clear *Pm* ortholog of the *Lf* cDNA fragments used as a probe/*LfHha* and was thus named *PmHha* (Fig. 1A, purple).

Contig 18919 (19.9 kb long, contains partial intron2 (2.3 kb) with a 107 bp sequencing gap and exon3) and contig 30618 (13.9 kb, contains only part of exon1 (87 bp)) were different in sequence from *PmHha* (kaki, Fig. 1A). They were however extremely similar to *LfHhb*, showing for example 92.7% identity with cosmid HH2 in their 5′ overlapping region (10.5 kb) and 97.8% identity in exon1 (partial sequence in contig 30618). Although we cannot prove that these two *Pm* contigs (30618 and 18919) are indeed from the same *Hh* locus, it is highly probable, based on the *Lf* genomic sequences. The existence of this locus is also supported by the finding of *PmHhb*-related exonic sequences in the trace archives of the *Pm* genome sequence project (kaki, Fig. 1A and phylogenetic tree in Fig. 2A). Contigs 18919 and 30618 therefore likely correspond to two short pieces of a *PmHh* locus orthologous to *LfHhb*, which we thus called *PmHhb* (kaki, Fig. 1A). Furthermore, BLAT search using *Lf* cosmids sequences retrieved up- and downstream sequences of the *PmHhb* genes. For example, the 5′ end of cosmid HH2 retrieved *Pm* contig18499 (15.0 kb) with 92.8% of similarity in 10.7 kb of overlapping sequence, which may therefore be assigned to be upstream sequence of the *PmHhb* locus (Figure S1A,B).

Finally, the contig 66870 contained sequences that spanned parts of intron1, exon2, intron2 (with a 1.3 kb gap) and exon3 of a *Pm* sequence highly similar but not identical to the *PmHhb* described above (Fig. 1A and Figure S2A). However, a stop codon was not identified in exon3 within the available sequence, and the sequence of the exon3 was highly divergent from the others, suggesting that this locus could be a pseudogene (Figure S2B). We named this putative *Hh* gene *PmHhb2*, with the possibility that this sequence may correspond to a *Petromyzon*-specific duplicate on the way of pseudogenisation.

As the *Pm* genome is not yet assembled, our *in silico* search cannot be considered as exhaustive and we cannot rule out the possibility that additional *Hh* gene(s) remain to be discovered. However, the parallel findings of two *Hh* sequences in two species of lampreys thought to have diverged 10–40 million years ago [2], using two different experimental approaches (*in silico* for *Pm* and *in vitro* for *Lf*) strongly support the existence of two *Hh* family members in the genomes of lampreys.

### Lamprey *Hh* genomic organisation

The overall organisation of lampreys *Hha* and *Hhb* are conserved with other species' *Hh* members, containing three exons and two introns (Fig. 1A). The first intron of *PmHha* is long (11 kb versus ∼5 kb in other studied species including *Lampetra*, and 3.2 kb in Fugu). Further sequence analysis detected many repeats corresponding to microsatellites. This phenomenon is coincident with the commonly accepted idea that *Pm* genome is rich in repeats, hence the difficulties in completing its assembly (University of Washington, http://genome.wustl.edu/genomes/view/petromyzon_marinus/).

Orthology relationships between *Hhs* in the two lampreys were well supported by cross-comparisons (Fig. 1C,D and Figure S2C). Nucleotide sequences were aligned and the percent identities between the four identified *Hhs* were calculated. The values presented in Figure 1C show remarkably high similarities between the orthologs, both in coding sequences (>95%) and within intronic regions (>91%, values in parentheses). Identities are much lower between paralogs, being only 60–74% in coding sequences and 50% in non-coding regions, and decrease to even lower values when cross-species/cross-gene comparisons are made (Fig. 1C,D).

### Lamprey Hha and Hhb belong to the Sonic/Indian group

Phylogenetic analysis was performed on the predicted amino-acid sequences of the exons. Neighbour-Joining (NJ) and Bayesian methods (together with Maximum Likelihood analysis shown in Figure S3B) all suggest that the two lamprey Hhs tend to cluster together within the Sonic/Indian Hedgehog clade (Fig. 2A and B; Figure S3C for a multiple alignment of the amino-acid sequences). These phylogenies are not very robust but the fact that all three types of analyses gave a similar tree topology favours the hypothesis that lamprey *Hh* genes indeed belong to the *Shh/Ihh* family. Of note, teleostean sequences seem to be rather fast-evolving, especially the *Ihh* and *Dhh* genes for which the monophyly with other *Ihh* and *Dhh* sequences is not well supported (e.g., Fig.2A, bootstrap = 32 and 54, respectively). Removal of these teleostean sequences from the alignment further reinforced the trend of lamprey *Hha* and *Hhb* genes to cluster with *Shh/Ihh* genes (Figure S3A). When included into the alignments, the amino-acid PmHhb sequence translated from trace archives (Fig. 1A, kaki) is the clear ortholog of LfHhb (Fig. 2A), and the partial LfHha sequence is the clear ortholog of PmHha (Fig. 2A and B). These data further support the relationships deduced from the genomic analysis and the existence of two Hhs in the two lamprey species.

This pattern is “classical” for lamprey genes, and has been reported in many studies for other genes/gene families for which orthology relationships are not easily inferred (e.g., for the *Otx* and other gene families [25], [39], [40]).

At the protein level, lamprey Hha and Hhb are almost identical in their hedgehog amino-terminal signaling domain (only 3 out of 150 amino-acids differ between the two sequences), a domain that is also extremely conserved in Shh and Ihh proteins of other species (Fig. 2C). By contrast, the Hedgehog/Intein domain (encoded by exon 3) is highly divergent between lamprey Hha and Hhb, as well as with Sonic/Indian sequences from other species (Fig. 2C). We took advantage of this feature to design *Hha*- and *Hhb*-specific probes (below).

### No *Dhh* in lampreys?

Our searches could not identify a *Desert*-like *Hh* gene in lampreys, which means that they either 1) have a *Dhh* that we have not found yet, 2) once had a *Dhh* but have lost it, or 3) have no *Dhh.* As it is clear that the cyclostome genomes have experienced at least one WGD –the event during which *Shh/Ihh* and *Dhh* genes arose from the ancestral *Hedgehog* gene [41], we favour the second possibility for two reasons. First, and as discussed above, we trust that the convergence of data obtained from two independent lamprey species and two independent search methods is a strong support for lampreys having only two *Hhs*. Then, if the two lamprey *Hhs* are more related to *Shh/Ihh*, the common ancestor of cyclostomes and gnathostomes must have had at least two genes, *Shh/Ihh* and *Dhh*. It is reasonable to think that *Dhh* has been lost in cyclostomes after their separation from gnathostomes.

Moreover, reasoning in terms of the function of *Dhh* and the emergence of novelties in the central nervous system of craniates is also attractive. Functional studies have shown that *Dhh* plays important roles in the development of peripheral nerves, controlling the formation of myelin nerve sheath [17]. Classical neuroanatomical studies also tell us that cyclostomes (both lampreys and hagfish) lack a myelin sheath around their axons, while all gnathostome brains including those of chondrychtyans like sharks and rays are indeed myelinated [42], making axon myelinisation a gnathostome novelty. A correlation between the emergence of myelin and the maintenance of a *Dhh* gene in gnathostomes is therefore striking, and myelinisation may be due to the recruitment of Dhh in a new function. A phylogenomics approach including synteny analysis will be helpful on these issues when *Pm* genome assembly is updated.

### *LfHha* and *LfHhb* expression patterns

Since the divergence of the sequences of lamprey *Hha* and *Hhb* genes suggest an ancient duplication of a *Shh/Ihh*-like gene, we next asked whether their expression would differ too. Previous analysis using a probe encompassing 276 bp of *LfHha* in the highly conserved hedgehog amino-terminal signaling domain (Fig. 2C for probe location) had revealed expression in the notochord at early stages and later in the floor plate of the neural tube, the hypothalamus and zli region of the ventral diencephalon, which appeared *Shh*-like [23]. This short probe was in a region of 93% identity (nucleotide level) between *Hha* and *Hhb*.

**Figure 3:**
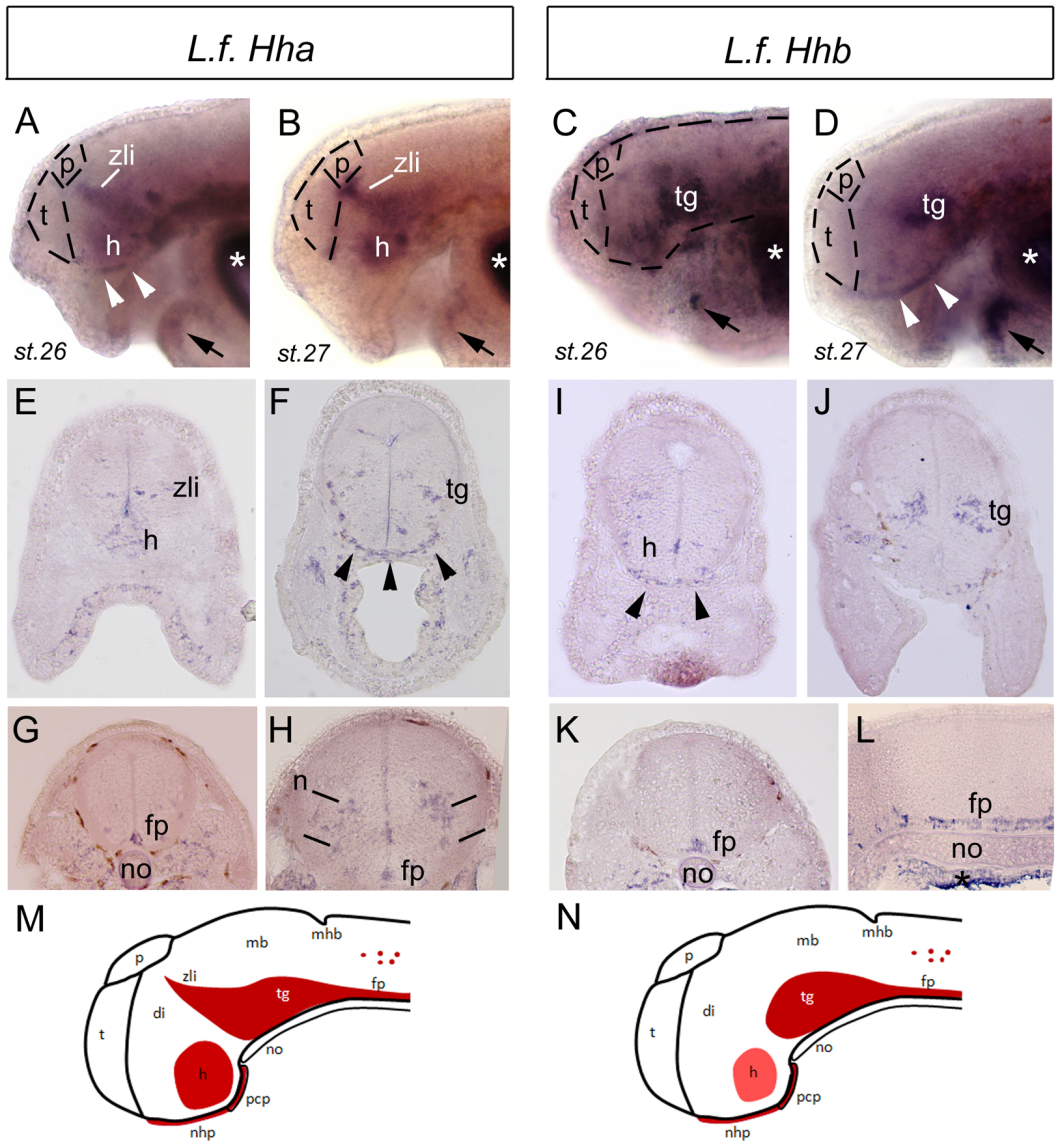
Compared expression patterns for *LfHha* and *LfHhb* in *Lampetra* embryos. **A-D** show *in toto in situ* hybridisation photographs for *LfHha* (A and B; left) and *LfHhb* (C and D; right). Stages are indicated. Anterior is to the left and dorsal is to the top. Dotted lines delineate the brain in A, and the telencephalon and pineal gland in A to D. **E-L** show *in situ* hybridisation photographs for *LfHha* (E-H; left) and *LfHhb* (I-L; right) on transverse sections (except L, saggital section) through the head of stage 27 *Lampetra* embryos. Sections are organised from the most anterior to the more posterior parts of the embryos. **M-N** show schematic summary drawings of the expression patterns for *LfHha* (M; left) and *LfHhb* (N; right), where *Hh* expression is red and the intensity of red is related to the expression density observed reproducibly on many sectioned embryos in independent experiments. In all panels, arrowheads indicate expression in a placodal structure identified as the nasohypophyseal placode (nhp) underlying the diencephalon in the continuity of the prechordal plate (pcp), arrows point to lower lip expression, and asterisks indicate background trapping in the branchial arches cavities. Abbreviations are: di, diencephalon; fp, floor plate; h, hypothalamus; p, pineal gland; pcp, prechordal plate; mb, midbrain; mhb, mid-hindbrain boundary; n, unidentified neuronal populations; nhp, nasohypophyseal placode; no, notochord; t, telencephalon; tg, tegmentum; zli, *zona limitans intrathalamica*.

In order to discriminate between *LfHha* and *LfHhb* transcripts, we generated paralog-specific probes containing mostly the third exon, which is the most divergent in sequence between *LfHha* and *LfHhb* (probe regions indicated on Fig. 2C). *In situ* hybridization performed on *Lampetra* embryos at stage 26 and 27, and analysed on whole-mounts (Fig. 3A–D) and on sections (Fig. 3E–L) showed that *LfHha* and *LfHhb* are expressed with very similar patterns. Expression was observed in the floor plate, the midbrain tegmentum, the hypothalamus and in scattered cell populations in the posterior neural tube. One important difference between the transcript distributions of the two genes is that *LfHha,* but not *LfHhb,* is expressed in the diencephalic zli organiser (summarised on Fig. 3MN). There is no *LfHh* expression in the basal telencephalon, which is the main difference between the lamprey *Hhs* and gnathostome *Shh*. Although we might have expected that *LfHhb* would be expressed in the telencephalon as a result of sub-functionalisation of the two paralogs in lamprey, it appears that this region of the embryonic forebrain is indeed devoid of Hh signalling. As we have suggested and discussed in previous studies, such differences in midline signalling systems between lampreys and gnathostomes may underlie the major neuro-anatomical differences observed in their telencephalon [23]–[25].

Importantly, we also observed previously unreported *LfHh* expression in the distal part of the lower lip and in a thin structure underlying the diencephalon ventrally, that we consider as a placodal structure and identify as the naso-hypophyseal plate/placode (nhp on Fig. 3MN). This finding is particularly interesting to discuss with regards to the developmental evolution of the adenohypophysis in lampreys. Uchida and colleagues [22] had proposed that duplication of *Bmp2/4*, hypothalamic expression of *Fgf8* and involvement of *Shh* in the hypophyseal placode were developmental novelties acquired in gnathostomes for the control of pituitary organogenesis. However, recent phylogenetic analyses suggest that the lamprey *Bmp2/4/16* family has well undergone duplications and secondary losses [43]; *Fgf8* expression in the lamprey hypothalamus is now demonstrated [25]; and here we found *LfHh* expression in the nhp. Thus, it appears that most of the key players involved in pituitary development were already recruited in the common ancestor of all vertebrates, in line with the idea that the lamprey hypothalamo-hypophyseal system displays strong similarities with its gnathostome homolog (e.g., [44]).

Both transcripts in lampreys are *Shh*-like in terms of their expression patterns. This feature, coupled to the poorly resolved orthology relationships seen in phylogenetic analyses (Fig. 2), leads us to propose that *Hha* and *Hhb* are lamprey duplicates belonging to the *Shh* family. To further test this hypothesis, we next performed a comparative genomic analysis of regulatory elements/CNEs between in lamprey *Hhs*.

### Are there any conserved non-coding elements (CNEs) in lamprey *Hh* genes?

**Figure 4:**
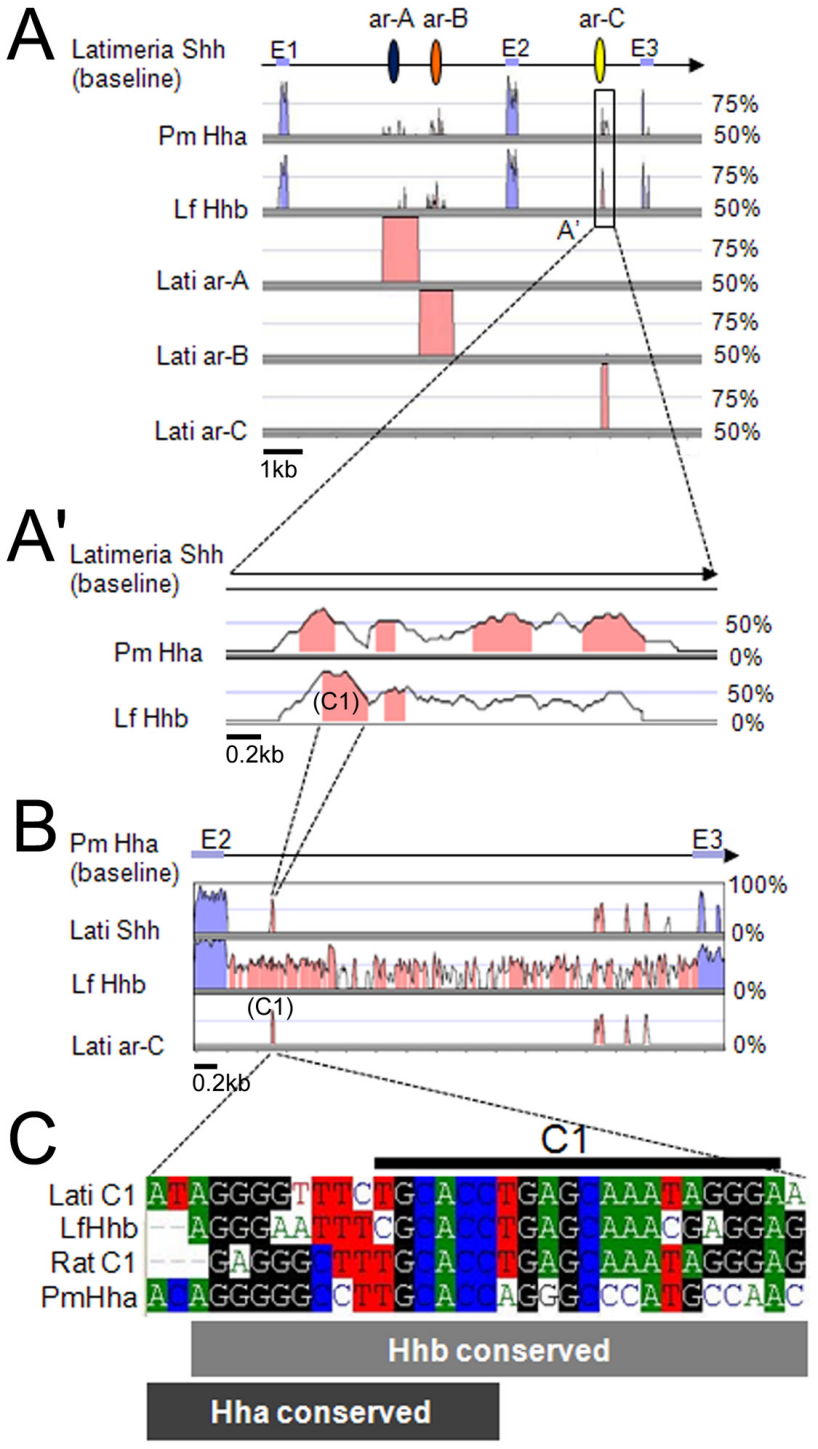
Conserved non coding elements (CNEs) in lamprey's *Hha* and *Hhb*. **A** is a search of CNEs in *PmHha* and *LfHhb* gene sequences, confined with the *Latimeria Shh* enhancers ar-A, ar-B, and ar-C. Both genes show conserved peaks in the putative enhancer regions. **A**' is a close-up on ar-C. **B** is a search of CNEs in intron2 using *LfHhb* as baseline, showing high conservation between lamprey *Hhs*, and dispersion of ar-C sub-elements throughout the *Latimeria* sequence. **C** is a direct visualization of the underlying nucleotide alignments between coelacanth, lampreys and rat *Shh/Hh* at the level of C1. Note that the motif conservation extends on the 5′ side in lamprey genes, and is particularly shifted in the case of *Hha*. Analyses were performed with the global alignment algorithm MLAGAN and visualized with VISTA plot, with Calc Window and Min Cons Width of 20 bp respectively, and 70% Cons Identity. In the baselines, purple boxes indicate the exon positions, while the blue, red, and yellow ovals are the positions of ar-A, ar-B and ar-C, respectively. Conservation peaks representing putative CNEs appear in pink.

Given the similarities in *Hh/Shh* expression patterns between lamprey and gnathostomes, it is challenging to identify functional CNEs shared in their genomes [24], [45]–[47]. The divergence and rapid evolution of teleost (zebrafish and *fugu*) *Hh* sequences observed in phylogenetic analyses prompted us to search for another reference genome to perform comparative genomic studies. We chose the coelacanth *Latimeria menadoensis*, a lobe-finned fish (sarcopterygian), which is considered, after the lungfish, as the closest living relative of tetrapods [14]. The coelacanth genome (as well as its phenotype) indeed seems more stable than any teleost genome, and it was proposed as a reference genome for the search for regulatory elements among gnathostomes using a comparative genomics approach [48]. Here we used the *Latimeria Shh* sequence (GenBank: FJ603040.1) as the base genome to perform the global alignments of lamprey intronic sequences in search of putative CNEs. Because the lamprey genome sequences are divergent from jawed vertebrate genomes, it was harder to align them with those of other vertebrates, although this type of alignment is reportedly easy to do between jawed vertebrate genomic sequences (e.g., [49], [50] (see also Figure S4). In order to identify putative CNEs, we added the sequences of *Latimeria* ar-A, ar-B, ar-C and ar-D identified by Hadzhiev, Lang and colleagues [37], [51] to our analysis. A global alignment using the LAGAN algorithm and visualization by VISTA plot easily identifies the three exons of lampreys *Hha/Hhb* and *Latimeria Shh* genes (blue peaks, Fig. 4A). Importantly, the CNEs of *Latimeria* can also be detected as significant conservation peaks in the intronic non-coding region of lamprey *Hh* genes (pink, Fig. 4A). Namely, ar-A and ar-B in intron1, and ar-C in intron2, can be identified in both *PmHha* and *LfHhb*. These findings indicate that the lamprey *Hha* and *Hhb* loci share CNEs with gnathostome *Shh* loci. Of note, when the same *in silico* analysis was performed using zebrafish *Shh* sequences as the baseline genome instead of *Latimeria*, less significant conservation hits were detected in the intronic regions (not shown), strengthening the usefulness of *Latimeria* as a reference genome.

### Lamprey *Hh* ar-C enhancer show homologous blocks with other ar-Cs

We next decided to focus on a single CNE, ar-C, whose conservation in sequence but not in function between zebrafish and mammals is quite puzzling [19], [21]. The conservation of ar-C between lampreys and *Latimeria* is the highest of the 3 enhancers (more than 75% for *LfHhb*, Fig. 4A and A'). It is also the *Hh* enhancer that has been analysed in greatest details [19], [20], [37], [52]. Previous functional analyses have subdivided the zebrafish ar-C element into four homology blocks, C1 to C4, which might be targets for transcription factors regulating the differential enhancer activities [37].

As shown on Figure 4A', a magnification of the lampreys ar-C conservation peaks identifies two to four highly conserved blocks (pink on Fig. 4A') for *LfHhb* and *PmHha*, respectively. Moreover and strikingly, VISTA visualisation of the global alignment of intron2 using a lamprey locus as baseline instead of the *Latimeria* locus also identifies four peaks of high conservation which correspond to *Latimeria* ar-C, but which appear dispersed throughout intron2 (Fig. 4B). This suggested that the *Latimeria* ar-C conserved elements are subdivided into short elements in lampreys. The most conserved peaks on Figure 4A' and 4B correspond to a C1-like nucleotide sequence, which is unusually located in the 5′ region of the lamprey intron2 (Fig. 4C and see below). These findings prompted us to test the possibility that lamprey ar-C contains the four homology blocks C1 to C4 described in other species, but that these blocks are dispersed or scattered along the entire intron2.

We then used *PmHha* and *LfHhb* ar-C sequences and checked for local alignment with *Latimeria* and rat ar-C sequences. The results show that both lamprey *Hhs* display homology blocks with other vertebrates in the C1 region, with the *LfHhb* C1 block displaying very good conservation, while the highly conserved C1 region in *PmHha* is slightly shifted towards 5′ (Fig. 4C). In addition, *PmHha* possesses a very short homology fragment within the C2 block, while *LfHhb* presents a well conserved block at a position corresponding to C3.

Ar-C of gnathostomes is commonly located in the 3′ region of intron2: in zebrafish *shha*, it is placed at positions 1035–1204 of the 1416 bp long intron2. In lampreys however, the putative ar-C1 region is located at the 5′ side of intron2, namely at positions 378–580 of the 3958 bp *PmHha* intron2 and at positions 759–959 of the 2589 bp *LfHhb* intron2. Our analyses demonstrate that putative functional modules can be detected *in silico* within lamprey CNEs, although they are significantly divergent from other vertebrates. In the following section, we therefore tested whether this conservation of sequence underlies functional conservation of these putative enhancers.

### Enhancer activity of lamprey ar-C and ar-C1

**Figure 5:**
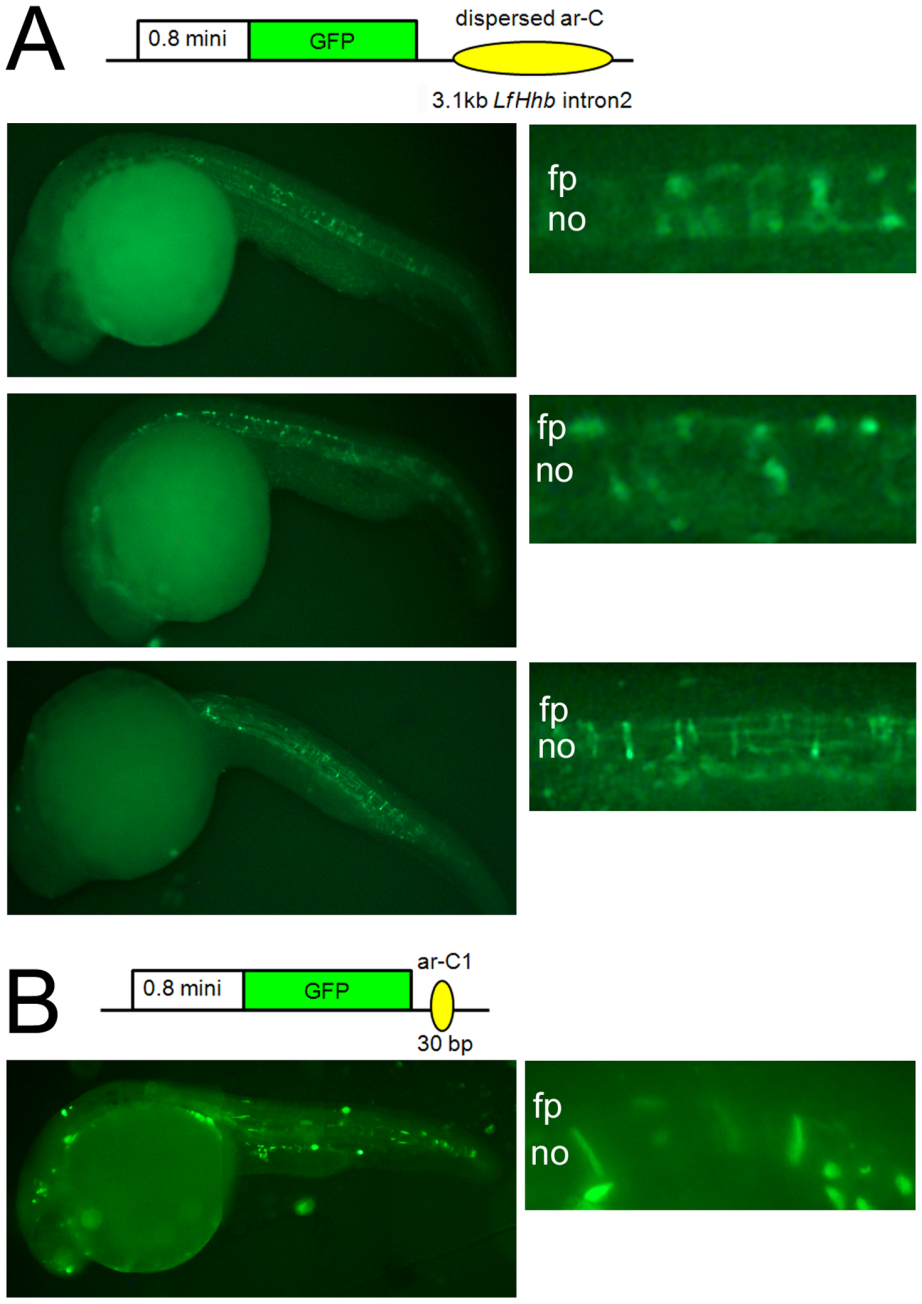
Functional analysis of lamprey CNEs in zebrafish embryos. **A**, *LfHhb* intron2 enhancer activity visualized in representative zebrafish embryos at 26hpf, at low (left) and high magnification (right) focusing on the notochord and floor plate. **B**, *LfHhb* ar-C1 enhancer activity visualized in two representative embryo at 26hpf, one at low (left) and one at high magnification (right) focusing on the notochord and floor plate. A schematic representation of the reporter constructs is shown in each case. It contains a zebrafish *shha* -0.8 kb minimal promoter driving GFP expression under control of the 3.1 kb intron2 (shown in A) or the 30 bp *LfHhb* ar-C1 (shown in B).

In zebrafish, *shha* expression at the ventral midline is mediated by ar-C [19], and C1 is crucial for ar-C activity [37]. Here, we have found that lamprey ar-C is dispersed along intron2, and that C1 conservation with gnathostomes is highest for *LfHhb* (Fig. 4). We therefore tested whether the entire intron2 of *LfHhb* has any enhancer activity. Injection of a GFP reporter construct in zebrafish embryos demonstrated that *LfHhb* intron2 drives GFP expression in the notochord and floor plate in 26hpf (hours post-fertilization) embryos (Fig. 5A and Table1). GFP was also observed in other ectopic tissues such as muscles, and considered as non-specific. The GFP reporter could be readily seen after observation in live embryos, and was further amplified for embryo scoring and photographing by immuno-fluorescence staining of the GFP protein (see also below). The backbone vector without any enhancer insertion gave almost no GFP expression, in agreement with previous works (Table 1; see also [20], [37].

**Table 1:** Quantification of GFP immuno-positive zebrafish embryos after reporter constructs injection at one-cell stage.

CONSTRUCT	GFP+ [nt/fp] (% of total/of GFP+)	GFP-positive [ectopic] (% of total/of GFP+)	GFP-positive [nt/fp & ectopic]	GFP-negative (including malformed embryos)	total number of injected embryos (n)
0.8Shh:GFP:LfHhb-I2	51 (10.1/32.9%)	104 (20.6/67.1%)	155 (30.6/−%)	350 (69.3%)	505
0.8Shh:GFP:LfHhb-C1	7 (3.7/15.6%)	38 (20.3/84.4%)	45 (24.0/−%)	142 (75.9%)	187
0.8Shh:GFP* (*negative control)	0 (0/0%)	10 (100/8.1%)	10 (8.1/−%)	123 (92.5%)	133

24–26hpf embryos injected with the indicated DNA construct were scored. nt:notochord; fp:floor plate.

Because the ar-C1 motif of ar-C seems to be the primary midline activator while other motifs seem to be repressors (ar-C2 and -C4) or of unknown activity (ar-C3) in zebrafish [37], we next tested the enhancer activity of *LfHhb* ar-C1 (Fig. 5B). The 30 bp-long *LfHhb* ar-C1 hardly had any detectable activity when observed *in vivo*, but clearly showed enhancer activity in the notochord after immuno-fluorescence staining. The global ratio of GFP-expressing embryos was almost the same as with the entire intron2 construct (24% versus 30%, Table 1). However, midline expression, including notochord and floor plate, was found with a ratio that was approximately half that observed with the entire *LfHhb* intron2 (15% versus 32% of the GFP-positive embryos, respectively, Table1). Furthermore, the relative intensity of GFP expression in the notochord appeared weaker than that driven by intron2. These data suggest that *LfHhb* C1 “midline” activity is significant, although weaker than that of the complete intron2.

Using a similar approach of phylogenetic footprinting, we have recently found that ar-C is shared and present in chordates *Hh* genes, but not in other deuterostomes [24]. Ar-C may therefore be a key motif for the chordate lineage, i.e., all the animals which possess a notochord and a floor plate.

### General conclusions

Animals share a great number of genes involved in developmental processes, and these are highly conserved in their coding regions, which points to their common and ancient origin, and to the strong selection against changes at amino-acid level in these proteins. Furthermore, CNEs are enriched and clustered around genes involved in developmental regulation. Yet there are few shared CNEs between invertebrates and other deuterostomes and even less related animals. It seems that developmental genes possess different CNEs and are under different regulation mechanisms in distantly-related species [53]–[55]. The comparison of the genome of a chondrichthyan, the elephant shark *Callorhinchus milii*, with other gnathostome genomes, indicated that the “vertebrate CNEs” had been fixed before the separation of chondrichthyans and other gnathostomes about 450–500 million years ago [56]–[58]. A recent report has identified CNEs in many amphioxus genes, but the single element identified upstream of *amphiHh* did not show significant enhancer activity *in vivo* in zebrafish [59]. In lampreys, CNEs were first found in *Hox* genes [45], [46], [60], and recently confirmed by the findings of McEwen et al.[47] who have surveyed 13 lamprey genes for CNEs, showing that some of them were functional in zebrafish, although they were less numerous than expected. Here, we have performed an in depth analysis of particular developmental genes, the *Hh* genes. Our findings emphasise the crucial role played by the evolution of developmental gene CNEs for the emergence of the vertebrate body plan. They also confirm that lampreys probably have a genome with all the “vertebrate-like” attributes, although with some degree of divergence. More data from other developmental genes will be necessary to confirm and generalize the case.

It is estimated that *Lampetra* and *Petromyzon* diverged from their common ancestor 10-40MYA [2]. Kuraku and Kuratani predicted that the genetic similarity between lamprey species within Petromyzoninae would be too high for phylogenomic footprinting. It is indeed the case. To our knowledge, our paper reports for the first time a genomic comparison between *Lf* and *Pm* introns, and it was not effective to detect or predict CNEs (data not shown; see also Fig. 4B). On the contrary, the high similarity in non-coding sequences found between the two species could be used as an additional argument to support the orthology between their respective *Hh* loci.

The identification of genomic sequences for two *Hh* genes in two species of lampreys enabled us to present a detailed description and analysis of the Hedgehog “family” (only two members) in an agnathan representative. Our findings that *Hha* and *Hhb* have no clear orthology relationship with their gnathostome counterparts (although they are clearly Shh-like in many aspects), that they structurally and functionally possess CNEs shared albeit somewhat divergent with other vertebrates (e.g., in terms of structural dispersion of the conserved motifs), and that their expression patterns have clear lamprey-specific features (an absence of telencephalic expression), suggest that the two lamprey *Hh* genes have evolved into a quite “lamprey-specific” way after the split between the last common ancestor of cyclostomes and gnathostomes.

## Supporting Information

Figure S1 PmContig18499 mapping in the 5'upstream region of the PmHhb locus using Lampetra cosmid sequences.
(0.08 MB DOC)

Figure S2 Detailed sequence comparisons and orthology assessments for lamprey Hh genes.
(0.39 MB DOC)

Figure S3 Additional phylogenetic trees
(2.82 MB DOC)

Figure S4 An example showing difficulty of global alignments of the lamprey genomic sequences with vertebrate sequences.
(0.15 MB DOC)
