# Supplementary material for: Two Lamprey Hedgehog Genes Share Non-Coding Regulatory Sequences and Expression Patterns with Gnathostome Hedgehogs
Source: PLoS One. 2010 Oct 13;5(10):e13332. doi: 10.1371/journal.pone.0013332 (PMC2954159; doi:10.1371/journal.pone.0013332)
Supplement: Figure S1 — PmContig18499 mapping in the 5'upstream region of the PmHhb locus using Lampetra cosmid sequences. (0.08 MB DOC) [file pone.0013332.s001.doc]

**Supplemental Figure S1: PmContig18499 mapping in the 5’upstream region of the *PmHhb* locus using *Lampetra* cosmid sequences.**

**(A) Mapping of the *Lf* cosmid HH2 on the current *Pm* genome assembly.**

**(B) Further Genome Annotation of *Pm* contig 18499.**


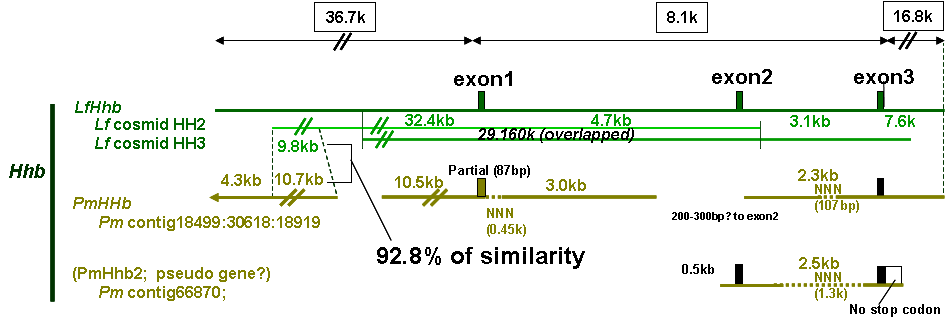


**(A)** Part of the *Lampetra* cosmid HH2 was additionally mapped on the PmContig18499 with 92.8% of sequence similarity using BLAT search implemented on the UCSC genome browser (<http://genome.ucsc.edu/cgi-bin/hgGateway?clade=other&org=Lamprey&db>=). Despite a high similarity of sequences, no coding sequence was identified in the overlapping genomic regions ranging from 7.3 to 9.8kb. Two major insertions of the *Pm* genome were observed when compared with the corresponding region of the *Lampetra* genome.

**(B)** Three independent *Petromyzon* contigs, 18499, 30618, and 18919, were assigned into a putative larger contig by referring to the *Lampetra* cosmid sequences. The corresponding regions revealed in (A) are indicated with two break lines between the *Lf* cosmid HH2 and *Pm* contig18499. This mapping allows us to survey wider genomic regions, approximately 60kb instead of 48.1kb using only the two *Lampetra* cosmids. As well, *Pm*contig22792 was assigned at upstream of *Pm*Contig4356 by referring to *Lf* cosmid HH4 as the lamprey *Hha* locus (data not shown). In this way, the two lamprey genomic resources can complement each other.
