# Supplementary material for: Two Lamprey Hedgehog Genes Share Non-Coding Regulatory Sequences and Expression Patterns with Gnathostome Hedgehogs
Source: PLoS One. 2010 Oct 13;5(10):e13332. doi: 10.1371/journal.pone.0013332 (PMC2954159; doi:10.1371/journal.pone.0013332)
Supplement: Figure S2 — Detailed sequence comparisons and orthology assessments for lamprey Hh genes. (0.39 MB DOC) [file pone.0013332.s002.doc]

**Supplemental Figure S2: detailed sequence comparisons and orthology assessments for lamprey *Hh* genes.**

**(A) Percent Identity Matrix of exon2 and exon3 among the lamprey *Hh* genes (at the nucleotide level)**

**Table A1. Comparison of exon2**

1: LfHha-exon2 100 98 93 93

2: PmHha-exon2 98 100 95 95

3: LfHhb-exon2 93 95 100 95

4: PmHhb2-exon2 93 95 95 100

**Table A2. Comparison of exon3**

1: LfHha-exon3 100 96 66 65 60

2: PmHha-exon3 96 100 63 62 52

3: LfHhb-exon3 66 63 100 95 56

4: PmHhb-exon3 65 62 95 100 56

5: PmHhb2-exon3 60 52 56 56 100

**(B) A Putative Lamprey *Hh* gene contained in the PmContig66870: amino acid translation in the 3 frames from the putative exon3 contained in the *Pm*Contig66870.**

>frame1

RTPSRPRAAAVSPATRASSRRAAAPRG*ATCGPASACWRRAAAPPRRPPTATSSSSSTAS

PASGAASSPWRRRAAAAFSSPRRTSCSPRPETPAAAAAVGPRRSAPWPSLPAACGLACSS

LSWRVLLLMLLLVVVGLVGLVVLVVVLVVVAVLMFLVLLVVVLVVVLVVLVVVVALILVV

VVLLVLLADVVVALVAIPLVVITRVVVLVVLLVLVSVMVVAMGVVVVVSLPKVVMMTVEA

VVVVVIFLIALLLVLLVPLVLTAVLALLTLPLVLPGLLLIPVRLFLSVPHASLPVAPIVF

RLLPAPLLVAFVLLVQLLVLLVQLLVLLRTLSLAALLMLVLFVPLLVLLPLAQLQVWPLA

LPVLSVRLLIQLRVLPPVPMTAPALAPPSLLRDRPPLMPAPLTERYGCX

>frame2

ELHRAQERRLFPRRLAPLHGGRRLQEAERPAARRARAGGERRRRLDARLQRPPPLPRPRA

PPAAPLRRPGDGGRRPPSRHPVAPRVRRARRLRRRRRRWGPGGARRGPLCQPRAAWHVRL

*AGGCSC*CCCWWWWGW*GWWCWWWCWWWWRC*CSWCCWWWC*W*CWWC*WWW*P*FWLW

WCCWCCWRTWWWHWWRYR*W*SQEWWCWWCC*SWCL*WSWQWGW*L*CRCRKW***Q*KR

WWWLLYFSSRCCSCCSCRSCLQLC*RC*HCHSFCRDCC*FQFDYFCRCHTHHCRWRPLFS

VCSRHRCWWRLFC*SSC*CCWFSCWSCCERFRWQRYSC*CCLFRC*FCCRWLSCRFGRWR

CRCCRFGC*FNCECCRLFR*RRRR*RRRRCCGIGRR*CRRR*RNATAA

>frame3 [a HINT domain predicted with PFAM is highlighted in yellow]

NSIAPKSGGCFPGDSRLFTEGGGSKRLSDLRPGERVLAASGGAASTPAYSDLLLFLDREP

RQRRRFVALETEGGGRLLVTPSHLVFAAPGDSGGGGGGGAPEERAVALFASRVRPGMFVF

ELAGALADAAAGGGGAGRAGGAGGGVGGGGGVDVPGVVGGGVSGSAGGVSGGGSLNFGCG

GAAGVVGGRGGGTGGDTASGDHKSGGVGGAASLGVCDGRGNGGGSCSVVAESGDDDSRSG

GGGCYISHRAAARAARAARAYSCASAVDIATRSAGTAANSSSTISVGATRITAGGAHCFP

FAPGTAAGGVCSASPAASAAGSAAGPAANAFAGSVTHASAVCSVASSAAAGSAAGLAAGA

AGAVGSAANSTASVAACSDDGAGASAAVAAAGSAAADAGAADGTLRL

**(C) Phylogenetic NJ tree using only exon2**


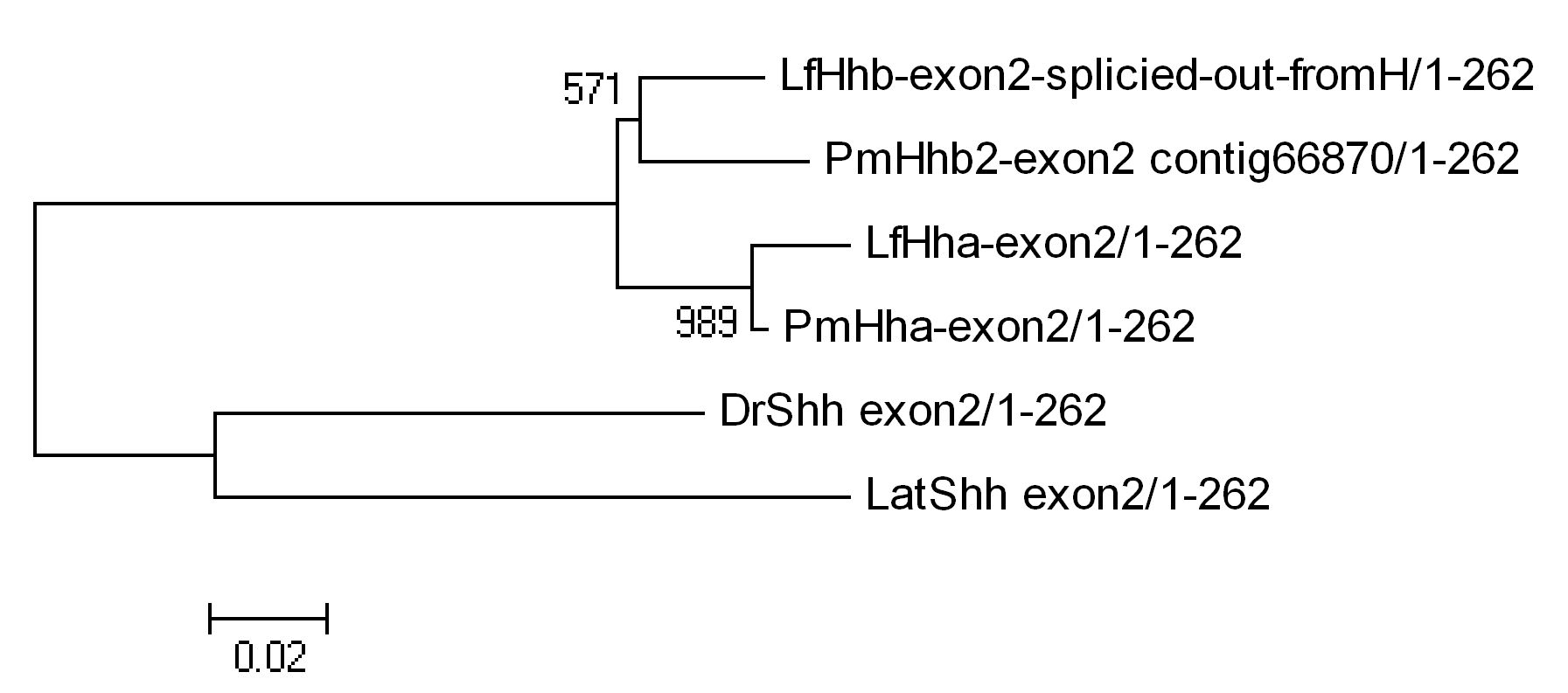


**(D) Nucleotide sequence alignment around a junction between intron2 and exon3 of the lamprey *Hh* genes**


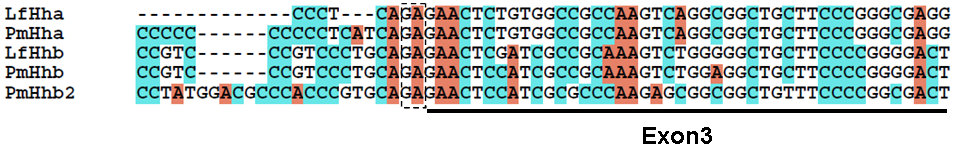


**(D’) Amino acid alignment for a part of exon3 of the lamprey Hh proteins**


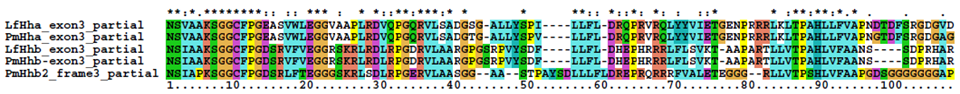


The Percent Identity Matrix, the NJ tree, and the alignment were obtained using the ClustalX software (version 2.0.10).

**(A)** Percent identity matrix at the nucleotide level. Each exon was separately compared since the exon3 is more divergent while exon2 is well conserved. Notably, exon3 of *PmHhb2* is divergent from the other lamprey *Hh* genes whereas orthologous genes are similar to each other (see percentages highlighted in green and blue).

**(B)** A NJ phylogenetic tree using only 262bp of exon2, which are well conserved among species including other vertebrates. *PmHhb2* is likely to be ortholog to *LfHhb* despite its relatively low bootstrap value, while it is apparently different from the lamprey *Hha* genes. **(C)** Amino acid residues in each frame translated from the exon3 of Pm Hhb2. Asterisks indicate stop codons. A HINT domain was identified in the frame3 of translation, which was identified with BlastX and Interpro scan (highlighted in yellow). The frame3, without the stop codon, results in at least 407 AA in length, which is longer than all other exon3 (approx. 300AA).

**(D and D’)** nucleotide and amino-acid sequence alignments around the junction between intron2 and exon3 of the lamprey *Hh* genes. A part of amino-acid sequences of exon3 containing the HINT domain. *PmHhb2* (bottom line) has apparently diverged from the other lamprey *Hh* genes in both non-coding (intron2, D) and coding sequences (D’).
