# Supplementary material for: Two Lamprey Hedgehog Genes Share Non-Coding Regulatory Sequences and Expression Patterns with Gnathostome Hedgehogs
Source: PLoS One. 2010 Oct 13;5(10):e13332. doi: 10.1371/journal.pone.0013332 (PMC2954159; doi:10.1371/journal.pone.0013332)

**Suppl. Figure 3 :**

A: HH NJ tree without teleostean sequences included.

B: HH ML (Maximum Likelihood) tree (50% condensed tree).

C: multiple alignment of the amino-acid sequences used to generates the trees (after removal of regions of ambiguous homology). The last 4 lines (indicated by asterisks) are the 4 lamprey Hh sequences.


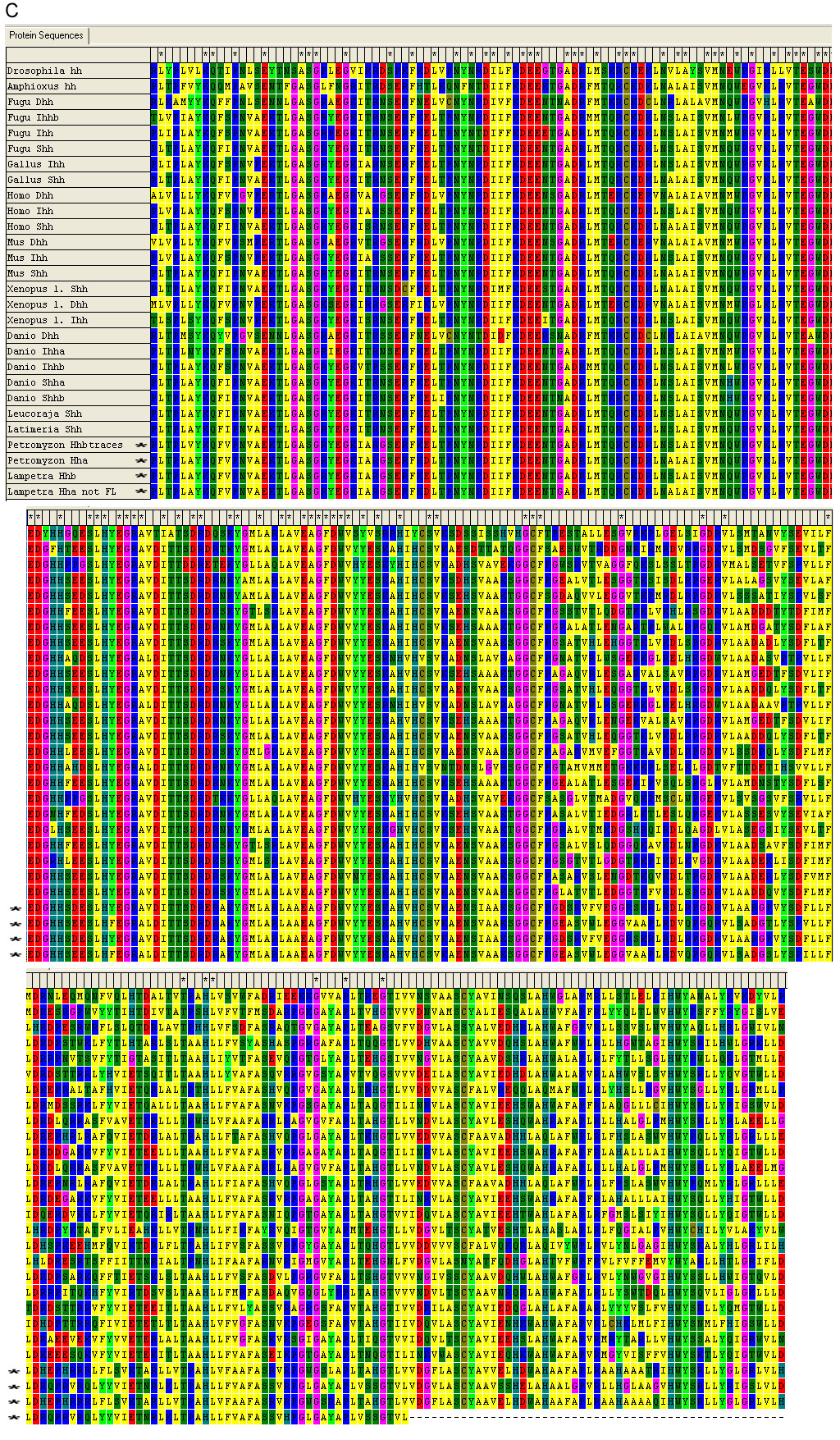

Supplement: Figure S3 — Additional phylogenetic trees (2.82 MB DOC) [file pone.0013332.s003.doc]
