# Supplementary material for: Two Lamprey Hedgehog Genes Share Non-Coding Regulatory Sequences and Expression Patterns with Gnathostome Hedgehogs
Source: PLoS One. 2010 Oct 13;5(10):e13332. doi: 10.1371/journal.pone.0013332 (PMC2954159; doi:10.1371/journal.pone.0013332)
Supplement: Figure S4 — An example showing difficulty of global alignments of the lamprey genomic sequences with vertebrate sequences. (0.15 MB DOC) [file pone.0013332.s004.doc]

**Supplemental Figure S4. An example showing difficulty of global alignments of the lamprey genomic sequences with vertebrate sequences.**


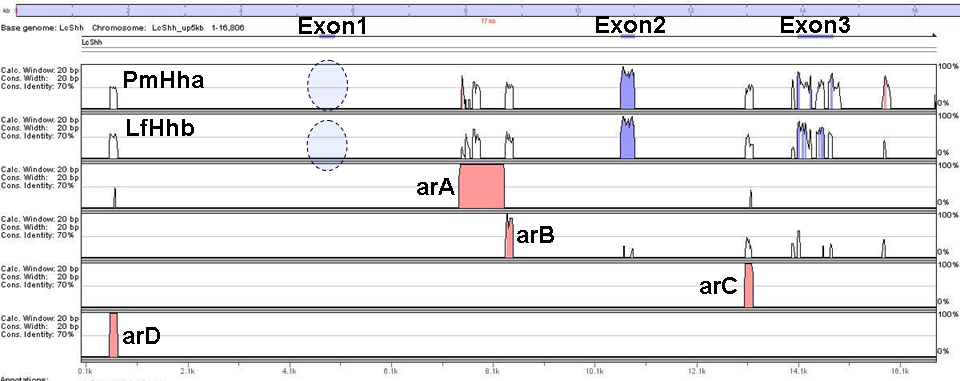


Entire genomic loci sequences were used for global alignments using MLAGAN and visualized using VISTA with the same parameters as those used in Figure 4 (the *Latimeria* *Shh* locus was used as the base genome). In this figure, exon1 does not appear (see circles in dooted lines), although exon2, exon3 and other known CNEs were identified. Use of only upstream sequences was successful in identifying both exon1 and arD as shown in Figure 4. Thus, tunes for global alignments are required to get better resolution of the lamprey genomes.
